# Testing the Glucose Hypothesis among Capuchin Monkeys: Does Glucose Boost Self-Control?

**DOI:** 10.3390/bs6030016

**Published:** 2016-08-03

**Authors:** Audrey E. Parrish, Ishara D. Emerson, Mattea S. Rossettie, Michael J. Beran

**Affiliations:** 1 Psychology Department, Georgia State University, Atlanta, GA 30302, USA; mberan1@gsu.edu; 2 Language Research Center, Georgia State University, Atlanta, GA 30302, USA; mrossettie@gsu.edu; 3 Department of Environmental Sciences, Spelman College, Atlanta, GA 30314, USA; iemerson@scmail.spelman.edu

**Keywords:** self-control, glucose, ego-depletion hypothesis, capuchin monkeys

## Abstract

The ego-depletion hypothesis states that self-control diminishes over time and with exertion. Accordingly, the glucose hypothesis attributes this depletion of self-control resources to decreases in blood glucose levels. Research has led to mixed findings among humans and nonhuman animals, with limited evidence for such a link between glucose and self-control among closely-related nonhuman primate species, but some evidence from more distantly related species (e.g., honeybees and dogs). We tested this hypothesis in capuchin monkeys by manipulating the sugar content of a calorie-matched breakfast meal following a nocturnal fast, and then presenting each monkey with the accumulation self-control task. Monkeys were presented with food items one-by-one until the subject retrieved and ate the accumulating items, which required continual inhibition of food retrieval in the face of an increasingly desirable reward. Results indicated no relationship between self-control performance on the accumulation task and glucose ingestion levels following a fast. These results do not provide support for the glucose hypothesis of self-control among capuchin monkeys within the presented paradigm. Further research assessing self-control and its physiological correlates among closely- and distantly-related species is warranted to shed light on the mechanisms underlying self-control behavior.

## 1. Introduction

Marathon runners condition their bodies across weeks of training, toning their muscles and strengthening their mind-set, all the while exhibiting impressive feats of physical and mental self-control and dedication in the effort to cross the finish line. According to the limited strength model of self-control, just as a marathon runner’s muscles fatigue after a long day of training, the runner’s self-control in choosing to stay committed to the effort also depletes in a similar fashion [1]. The strength model of self-control, also known as the limited resource or ego-depletion hypothesis, states that self-control diminishes with each exertion or expression of such control, such that there are subsequently fewer resources available for future circumstances requiring self-control (e.g., [2,3]). Early research documented compromised self-control performance among individuals participating in a series of un-related self-control tasks, such that fewer resources were available for tasks occurring later in the sequence. For example, in the earliest ego-depletion study using the dual-task paradigm, participants were either instructed to regulate their emotions (or not) while watching an emotional film. Those participants who exercised self-control to regulate their emotions during the film performed poorer on a subsequent self-control measure of physical strength (stamina during a handgrip task) than participants who did not suppress their emotions [4]. Relatedly, researchers documented compromised performance on an unsolvable puzzle task after participants exercised self-control to avoid eating chocolates in favor of a less preferred but healthier food option (radishes) than those who did not suppress their urge to eat the chocolate [1]. A recent meta-analysis of 83 studies reported significant effect sizes for ego depletion on participant effort, perceived task difficulty, negative affect and fatigue, and blood glucose [5]. Following the early research on the ego-depletion model, researchers sought out the energy source for “willpower” in an effort to resolve what allows some people to exercise self-control in some circumstances but fail to do so in others.

The glucose hypothesis posits this state of diminished resources following self-control exertion is linked to decreases in blood glucose (see [6,7] for reviews). Crucial to this hypothesis, glucose is considered as the fuel for the brain and is implicated in a suite of effortful cognitive processes, such as executive functioning, memory processes, and attention control (e.g., [8,9,10,11,12,13,14,15,16,17,18]). Moreover, decrements in glucose have been linked to a wide variety of self-control failures, including attention control, emotion regulation, aggression and violence, alcohol and tobacco use, and social impulsivity (see [6] for a review). Experimental increases of circulating blood glucose (via glucose injections and high-sugar meals or drinks) and even simply tasting glucose (without ingestion) enhances self-control performance in some tasks (e.g., [19,20,21,22]; for a review see [7]). Conversely, decreases in blood glucose (via insulin-dependent diabetes mellitus) are associated with diminished self-control performance (e.g., [23,24]).

Despite the large number of studies assessing the ego-depletion model of self-control and its link to glucose, a recent meta-analysis correcting for small study effects revealed little evidence that self-control relies upon a limited resource [25]. Further, there are a growing number of studies that have failed to find support for the link between self-control and blood glucose, complicating the story of a simple physiological mechanism underlying self-control (e.g., [26,27,28,29,30]). Relatedly, attempts to shed light on the mechanisms underlying self-control in distantly- and closely-related species have yielded contradictory findings. For example, a recent study with capuchin monkeys revealed limited support for the ego-depletion hypothesis and no evidence for the link between glucose and self-control performance [31]. Monkeys were given the accumulation test of self-control [32] which presents food items one-by-one to a subject until the subject retrieves and eats the items, allowing for spontaneous measures of self-control as an animal inhibits taking earlier items in a food set to facilitate the accumulation of additional items over time. Subjects performed the self-control accumulation task pre- and post-breakfast, following a nocturnal fast to examine the effects of glucose-depletion on self-control [31]. Accumulation performance was measured pre- and post-high carbohydrate breakfast to test the effects of energy depletion on self-control. Additionally, accumulation performance was measured following a simple cognitive task (computerized touching task) versus a more demanding cognitive task (computerized matching-to-sample task) to examine the effects of cognitive-depletion on self-control. Self-control performance decreased over trials within testing sessions (there were three accumulation trials per session), but did not improve following a glucose-enriched breakfast meal. Self-control also was not affected by performing a more demanding computerized cognitive task prior to the self-control test. These results provided only partial support to the limited strength model [2], such that self-control of capuchin monkeys diminished slightly across repeated tasks. However, these results were not consistent with the glucose hypothesis in that the monkeys’ self-control performance was not increased from a boost in glucose following meal consumption. 

Intriguingly, self-control among more distantly-related species to humans has improved following diet-related glucose increases. Self-control among dogs (*Canis familaris*) on an unsolvable foraging task was compromised if the dogs had just completed a control task requiring subjects to remain in the “stay” position for 10 min [33]. This depletion effect was eliminated in a follow-up experiment for dogs that were administered a glucose-enriched drink prior to the first task. Relatedly, dogs demonstrated higher working-memory performance in a visible displacement task with hidden food following a self-control task given to the dogs 30 min after a meal versus dogs tested in a fasted state [34]. When presented with the smaller-sooner/larger-later inter-temporal choice self-control paradigm, glucose-depleted and starved honeybees preferred an immediate, but less-preferred option to a delayed, but better option following a 24-h fasting period [35]. Further research is necessary to understand these contradictory findings within and across species, and whether a simple physiological mechanism such as glucose ingestion can support widespread and sometimes complex self-control processes. 

In the current study, we assessed the glucose hypothesis among capuchin monkeys using the accumulation paradigm as our measure of self-control. We compared monkey performance on the accumulation task after a nocturnal fast and either a glucose-enriched breakfast meal or a low glucose breakfast meal. A limitation of the De Petrillo et al. [31] study with capuchin monkeys was that it did not provide a control condition in which the caloric intake was equated independent of the delivery of glucose. In our approach, the low glucose meal in the current experiment was matched for calories to the glucose-enriched meal to separate the effect of glucose from satiation on self-control performance. Crucially, a calorie-matched control meal allowed us to investigate the effects of glucose on self-control in the absence of differential motivational factors such as hunger or motivation. The resulting data will help clarify the relation of glucose consumption to self-control, and provide further insights into the value of comparative models for assessing this hypothesis. 

If the glucose hypothesis holds for capuchin monkeys in this self-control procedure, we predicted that accumulation performance would increase in trials following the high glucose meal versus trials following the control meal. We anticipated individual differences in capuchin monkey performance based off of previous testing with these same subjects [36,37,38].

## 2. Materials and Methods

### 2.1. Subjects

We tested nine adult capuchin monkeys, sometimes referred to as *Cebus apella* or as *Sapajus apella* [39,40], four females (ages 13 to 18 years) and five males (ages 7 to 17 years), from Georgia State University’s Language Research Center in Atlanta, GA, USA. All monkeys were group-housed with conspecifics in indoor/outdoor enclosures with enrichment (i.e., climbing structures, swings, toys, browse) available. Monkeys voluntarily separated for solo testing during which they had visual and auditory access to group mates. They received a daily diet of chow, fruits and vegetables and were never food deprived or weight reduced for testing purposes. Monkeys were not fed overnight for the glucose manipulation in the current study, but note that this is their routine feeding schedule and we did not deviate from the animal’s typical dietary routine. Water was available ad libitum. Testing complied with the procedures and protocols that were approved by the Institutional Animal Care and Use Committee of GSU (protocol A13014). GSU is accredited by the Association for Assessment and Accreditation of Laboratory Animal Care. 

All monkeys had participated in several self-control studies assessing self-control performance on the accumulation task [36,37,38]. Thus, they came into the experiment with relevant experience regarding the accumulation paradigm. 

### 2.2. Apparatus

**Figure 1:**
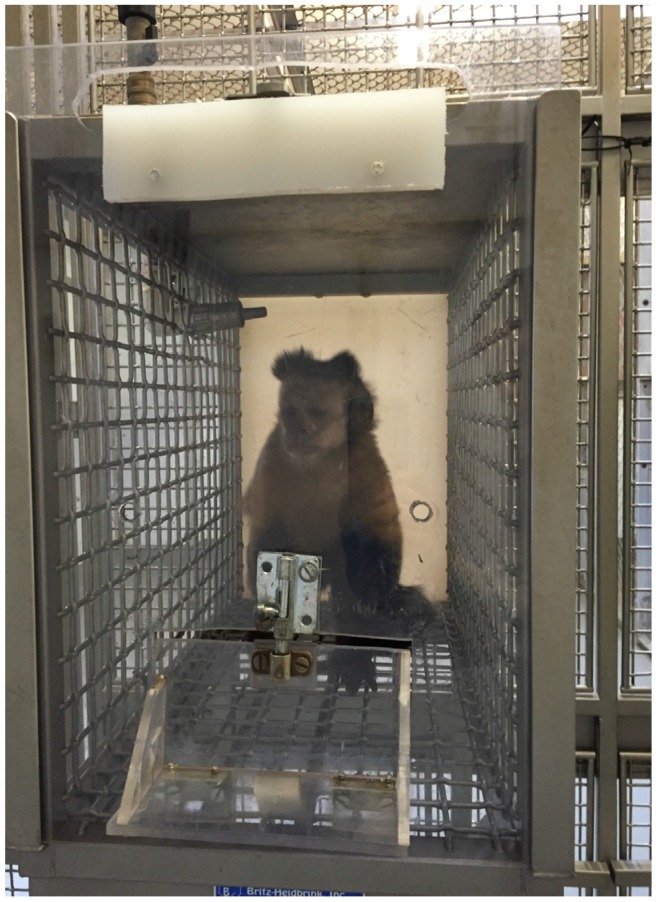
Testing apparatus.

Monkeys voluntarily entered and were tested in individual stainless steel mesh test boxes (33 cm × 46 cm × 61 cm) that were attached to the group enclosure. A vertical Plexiglas panel was attached to one end of the test enclosure. A hinged Plexiglas pan (15 cm × 7.5 cm) was attached to the front of the panel so that its contents were accessible by the monkey (if the pan was hinged inwards) and by the experimenter (if the pan was hinged outwards). A deadbolt lock could be used to lock the pan in the outwards position so that only the experimenter could access its contents (see Figure 1). 

### 2.3. General Procedure

Testing began at approximately 10:00 AM–11:30 AM, 2–3 days per week (one session per day). As part of the normal feeding routine, monkeys were not provided any foods overnight for a 12 h time period (9:00 PM to 9:00 AM) prior to testing. Note that these animals participated in the current test as their first study in the day, but the capuchin monkeys were awake for a minimum of two to three hours prior to testing. At approximately 10:00 AM the following morning, monkeys received a glucose-rich meal (High Glucose Condition) or a low-glucose calorie-matched meal (Low Glucose Condition) prior to the accumulation phase. The High Glucose meal consisted of honey-roasted peanuts and raw honey. The Low Glucose meal consisted of un-salted peanuts, and one honey roasted peanut consumed last to ensure that the monkeys had the taste of the sweetened food present in each condition to prevent cuing the animals as to the condition. The caloric intake and sugar magnitude for both conditions varied across animals based on weight and condition (Table 1). All animals consumed the full meal for both conditions prior to each testing session. The accumulation test phase began 25–30 min following meal consumption (average of 28 min for both conditions). 

Monkeys completed one forced-accumulation trial followed by one free-accumulation trial per session. In the forced-accumulation trial, the pan was locked outwards so that the monkeys could not access its contents until the experimenter unlocked the pan. The experimenter transferred five raisins into the pan from a transparent bowl at a 2 s delivery rate. The experimenter then unlocked the pan, which gave monkeys access to the accumulated rewards. Total-trial length for the forced-accumulation trial was 60 s, immediately followed by the free-accumulation trial. The purpose of the forced-accumulation trial was to remind the monkey of the nature of the accumulation procedure and the inter-item delivery rate. In the free-accumulation trial, the pan deadbolt was left unlocked so that the monkey could access its contents at any time. The experimenter transferred up to 50 raisins from a transparent bowl every 2 s until the monkey consumed a reward. After the monkey retrieved and consumed a reward from the pan, the experimenter refrained from adding additional items to the pan, effectively ending the accumulation procedure.

Each monkey completed 20 sessions—10 of each condition (10 High Glucose sessions and 10 Low Glucose sessions). Monkeys participated in only one session per day, experiencing one High Glucose condition and one Low Glucose condition within every two sessions completed. The order of the condition within these two sessions was randomized (i.e., monkeys sometimes completed the High Glucose condition prior to the Low Glucose condition and vice versa per two-session block).

## 3. Results

**Figure 2:**
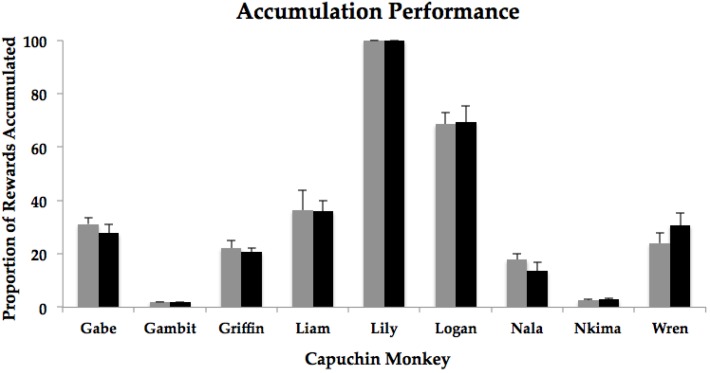
Accumulation performance for individual capuchin monkeys for the Low Glucose (gray bars) and High Glucose (black bars) conditions. Bars represent the mean proportion of rewards accumulated in each condition. Error bars depict standard error of the mean.

We first conducted a paired-samples t-test to compare accumulation performance as measured in terms of total number of items accumulated between the Low Glucose condition (*M* = 16.93, *SD* = 15.88) and the High Glucose condition (*M* = 16.84, *SD* = 16.01), *t*(8) = 0.17, *p* = 0.87. Because of the large individual differences in performance, we also conducted individual paired t-tests for each monkey. There were no differences for any monkey in the total number of raisins accumulated between conditions, all *p* values > 0.05 (Table 2 and Figure 2). See Table 2 for information regarding the mean number of raisins accumulated by each subject in the two conditions. Note that one monkey (Lily) accumulated all raisins in both conditions, but in one High Glucose condition session, only 49 raisins (instead of 50) were offered for accumulation due to experimenter error.

To assess changes in accumulation performance as a function of increasing experience with the task across sessions, we calculated the correlation of session number and total number of raisins accumulated for both conditions for each monkey (two-tailed test; Table 3). Performance remained consistent throughout the study for nearly all animals in both conditions. However, one monkey (Nkima) increased his performance within the High Glucose condition from an average of one raisin to two raisins over the course of the ten sessions. Two monkeys’ performances decreased over the course of testing (Wren—High Glucose and Liam—Low Glucose). 

## 4. Discussion

If glucose ingestion provides fuel for self-control, the present manipulation should have led to increases in accumulation among capuchin monkeys when glucose was present in the first meal of the day versus when it was not present following a nocturnal fast. However, accumulation performance of these monkeys did not differ between glucose conditions. These results are consistent with assessments of potential depletion effects that do not report an impact of glucose on self-control performance [26,27,28,29,30].

Interestingly, De Petrillo and colleagues [31] reported partial support for the ego-depletion hypothesis, as capuchin monkey performance on their accumulation task decreased over trials within a session, suggesting decrements in self-control via prior exertion. However, the reported decrement was limited; performance decreased across three test trials within a session from an average of approximately three accumulated food items to two items. However, consistent with the current findings, they did not report increases in self-control performance following a glucose-enriched meal. Here, our goal was to assess a link between basic performance levels in the accumulation task as we typically employ it (with only one or a small number of trials per session) and glucose ingestion. Thus, we administered only one test trial per session, and could not measure within-session changes in self-control. Our results demonstrated consistent performance by capuchin monkeys across testing sessions, with little variance at the individual level, and crucially, across glucose conditions. However, future studies implementing the dual or sequential task paradigm (a series of self-control tasks within a given testing session: 1,4) to nonhuman primates would address the ego-depletion hypothesis as it is typically assessed within the human literature in conjunction with the glucose hypothesis.

For human adults, the link between self-control and glucose does not hold with the introduction of highly motivating rewards (e.g., cash rewards; [41]), a behavior that may be adaptive given the incentives available for maintaining high self-control performance in the face of depleted resources [6]. Monkeys accumulated a preferred food item (raisins) in the current study, which may have been sufficiently motivating for the animals such that any effect of glucose-depletion was overridden. However, this seems unlikely, given that most animals did not approach high levels of accumulation, a finding in stark contrast to that seen in humans. Future self-control tasks that incorporate low-valued rewards or non-food based measures of performance among nonhuman animals are necessary to test this hypothesis more completely. However, this task is not without challenge for nonhuman subject testing, as food-based self-control measures are the standard in animal testing to motivate engagement in the presented tasks.

Following previous study designs assessing the glucose hypothesis in humans and nonhuman animals (e.g., [18,31]), we manipulated glucose levels in the early part of the day. By testing animals in the morning, after an overnight period with no food, we had the best possible control of the glucose levels of these animals given that in our laboratory we do not food restrict or water restrict any of the animals. Thus, the use of the morning testing best allowed for glucose to be greatly increased in the first meal monkeys received for the day relative to a calorie-matched control meal. Previous research with human children documented enhanced performance on attention and memory tasks following a breakfast meal that was high in complex carbohydrates, with a greater effect several hours following breakfast [18]. It is important to note that we did not document precisely how long the monkeys had been awake prior to testing, but the animals were awake for a minimum of two to three hours prior to the breakfast meal as part of their normal husbandry routine. Future studies that assess time-of-day effects within this species are necessary to establish when glucose (or other dietary substitutions) might have the greatest impact on self-control and other cognitive capacities. 

Given the non-invasive nature of this study, we did not establish physiological measures of blood glucose levels for the monkeys pre- and post-meal. Also, we presented one honey peanut at the end of the low-glucose meal to avoid cuing the animals as to which condition would be presented following the breakfast meal. Although this occurred thirty minutes prior to testing and there was a vastly larger amount of glucose present in the high-glucose meal than the low-glucose meal, previous studies have documented that simply tasting glucose can lead to increases in self-control [20,21]. Thus, future studies that present a calorie-matched meal with no glucose are critical to establishing whether the consistent performance across conditions observed in the current study is perhaps a byproduct of the significantly lower, but not non-existent glucose in our control condition. Despite this, our method of testing self-control performance following a fast is a commonly used practice with humans and nonhuman animals. This method has produced varying degrees of success in manipulating self-control performance (e.g., [18,34]). Additionally, the monkeys in the current study were familiar with the accumulation paradigm, participating in several studies using this self-control assessment [36,37,38]. It is possible that the effects of glucose on self-control are slight, and may emerge under novel testing conditions and spontaneous instances of self-control. However, this seems unlikely given that most monkeys were not near ceiling levels of performance, and thus could have shown improved self-control through greater accumulation after glucose ingestion if that ingestion strengthened such self-control. Future research measuring exact blood glucose levels pre- and post-meal and with task-naïve participants is important to quantifying the effect of glucose on self-control and in delineating the role of practice or expertise in the ego-depletion and glucose hypotheses.

The present results argue against a link between glucose and self-control from a comparative perspective, at least when methods to deplete such self-control are not employed. In tightly controlled and matched conditions, a vastly larger dose of glucose delivered to monkeys that were fasted overnight in their normal dietary routine did not evoke any hint of increased performance in waiting for a better outcome of more rewards. As such, for the accumulation task with these monkeys, there appeared to be no link between glucose ingestion and self-control. However, other species need to be tested, other tasks need to be included, and other variations of depleting self-control, providing glucose and measuring glucose levels at test time need to be employed. Until that time, the present results coupled with previous findings among the comparative and human literatures suggest caution about linking self-control and glucose within nonhuman primates.

## 5. Conclusions

The ability to forego something immediately rewarding in favor of a delayed, but larger or more enticing reward reflects one’s self-control. A potential physiological mechanism underlying this capacity has been proposed through the glucose hypothesis, which states that depletions in self-control are reflective of depletions in blood glucose levels [6,7,13]. The current study did not support a link between glucose and self-control in capuchin monkeys as assessed in the accumulation task. Future research using alternative self-control paradigms and alternate physiological measures of glucose metabolism could shed further light on the mechanisms underlying self-control capacities in nonhuman primate species.

## Figures and Tables

**Table 1:** High Glucose and Low Glucose meal details.

		High-Glucose Condition				Low-Glucose Condition		
Monkey	Weight (kg)	Honey Peanuts (g)	Honey (g)	Sugars (g)	Calories	Unsalted Peanuts (g)	Sugars (g)	Calories
Gabe	3.6	3	9	7.29	41.8	8	0.43	42.9
Gambit	2.61	3	7	5.76	36.1	7	0.39	37.5
Griffin	5	3	12	9.57	50.4	9	0.47	48.2
Liam	3.82	3	9	7.29	41.8	7.5	0.41	40.2
Lily	3.51	3	8	6.53	38.9	7	0.40	37.5
Logan	4.1	3	10	8.05	44.6	8.5	0.45	45.5
Nala	3.17	3	7	5.76	36.1	7	0.40	37.5
Nkima	3.67	3	9	7.29	41.8	7.5	0.41	40.2
Wren	2.33	3	5	4.24	30.4	5.5	0.34	29.5

**Table 2:** Individual results for accumulation performance in the High Glucose and Low Glucose conditions. Paired-samples t-test results (High Glucose condition versus Low Glucose condition).

Monkey	Mean Number of Accumulated Raisins		T-Test Results
	Low Glucose	High Glucose	
Gabe	15.6	13.9	*t*(9) = −1.48, *p* = 0.17
Gambit	1	1	NA *
Griffin	11.1	10.3	*t*(9) = −0.44, *p* = 0.67
Liam	18.2	18	*t*(9) = −0.10, *p* = 0.92
Lily	50	49.9	*t*(9) = −1.00, *p* = 0.34
Logan	34.3	34.7	t(9) = 0.09, *p* = 0.93
Nala	8.9	6.9	*t*(9) = −1.55, *p* = 0.16
Nkima	1.3	1.5	*t*(9) = 1.00, *p* = 0.34
Wren	12	15.4	*t(*9) = 1.54, *p* = 0.16

* Gambit accumulated the same number of rewards throughout both conditions, and thus no test was conducted.

**Table 3:** Individual correlation results (session number and accumulation performance) to assess the overall role of experience on accumulation performance throughout the course of testing.

Monkey	Correlation Results	
	Low Glucose	High Glucose
Gabe	*r(*10) = 0.42, *p* = 0.23	*r*(10) = 0.53, *p* = 0.12
Gambit	NA *	NA *
Griffin	*r*(10) = −0.43, *p* = 0.22	*r*(10) = 0.24, *p* = 0.51
Liam	*r*(10) = −0.67, *p* = 0.03	*r*(10) = −0.40, *p* = 0.25
Lily	NA *	*r*(10) = 0.17, *p* = 0.63
Logan	*r*(10) = −0.28, *p* = 0.43	*r*(10) = −0.33, *p* = 0.35
Nala	*r*(10) = −0.41, *p* = 0.24	*r*(10) = −0.29, *p* = 0.42
Nkima	*r*(10) = 0.27, *p* = 0.46	*r*(10) = 0.87, *p* = 0.001
Wren	*r*(10) = −0.42, *p* = 0.23	*r*(10) = −0.80, *p* = 0.006

* Gambit accumulated the same number of rewards throughout all session in both conditions and Lily accumulated the same number of rewards throughout the Low Glucose condition.
